# Moving beyond individual barriers and identifying multi-level strategies to reduce anemia in Odisha India

**DOI:** 10.1186/s12889-020-08574-z

**Published:** 2020-04-06

**Authors:** Erica Sedlander, Michael W. Long, Satyanarayan Mohanty, Ashita Munjral, Jeffrey B. Bingenheimer, Hagere Yilma, Rajiv N. Rimal

**Affiliations:** 1 Department of Prevention and Community Health, Milken Institute School of Public Health, The George Washington University, 950 New Hampshire, Washington D.C., 20052 USA; 2 DCOR Consulting, Bhubaneswar, Odisha India; 3 IPE Global, Delhi, India; 4 Department of Health, Behavior and Society, Johns Hopkins Bloomberg School of Public Health, Baltimore, USA

**Keywords:** Iron deficiency anemia, Social norms, Gender norms, India

## Abstract

**Background:**

To reduce the prevalence of anemia, the Indian government recommends daily iron and folic acid supplements (iron supplements) for pregnant women and weekly iron supplements for adolescents and all women of reproductive age. The government has distributed free iron supplements to adolescents and pregnant women for over four decades. However, initial uptake and adherence remain inadequate and non-pregnant women of reproductive age are largely ignored. The aim of this study is to examine the multilevel barriers to iron supplement use and to subsequently identify promising areas to intervene.

**Methods:**

We conducted a qualitative study in the state of Odisha, India. Data collection included key informant interviews, focus group discussions with women, husbands, and mothers-in-law, and direct observations in health centers, pharmacies and village health and nutrition days.

**Results:**

We found that at the *individual* level, participants *knew* that iron supplements prevent anemia but underestimated anemia prevalence and risk in their community. Participants also *believed* that taking too many iron supplements during pregnancy would “make your baby big” causing a painful birth and a costly cesarean section. At the *interpersonal* level, mothers-in-law were not supportive of their daughters-in-law taking regular iron supplements during pregnancy but husbands were more supportive. At the community level, participants reported that only pregnant women and adolescents are taking iron supplements, ignoring non-pregnant women altogether. Unequal gender norms are also an upstream barrier for non-pregnant women to prioritize their health to obtain iron supplements. At the *policy level,* frontline health workers distribute iron supplements to pregnant women only and do not follow up on adherence.

**Conclusions:**

Interventions should address multiple barriers to iron supplement use along the socio-ecological model. They should also be tailored to a woman’s reproductive life course stage: adolescents, pregnancy, and non-pregnant women of reproductive age because social norms and available services differ between the subpopulations.

## Background

Iron and folic acid supplements (IFA) are a safe and effective method to prevent anemia, particularly in low-income countries where regular consumption of iron rich foods may be financially out of reach [5]. IFA are particularly critical in India where anemia affects 53% of women between 15 and 49 years old [18]. Given these high rates, in 2012, the World Health Assembly Resolution endorsed six Global Targets for 2025, with its second target aiming for a 50% reduction of anemia in women of reproductive age (WRA) [44]. Nationally, the Indian government has been promoting and distributing free IFA for pregnant and lactating women since 1970 [21]. Since 2000, the Adolescent Girls Anemia Control Program provides weekly IFA supplements to adolescent girls at school [1, 40]. Later in 2013, the government adopted a life-cycle approach to address the problem of Anemia under the National Iron Plus Initiative (NIPI), which recommends weekly iron supplements for all women of reproductive age [24]. Most recently, in 2018, the government rolled out the Intensified National Iron Plus Initiative “Anemia Mukt Bharat” [17].

However, despite IFA guidelines, non-pregnant women of reproductive age (WRA) are largely ignored in national outreach efforts and in the literature. Non-pregnant women are an important sub-population to consider; although young children and pregnant women have the highest rates of anemia, non-pregnant women make up the greatest number of individuals with anemia [18]. Additionally, iron deficiency before a women gets pregnant can be difficult to replete once she is pregnant [41]. The vast majority of women in India with anemia have mild-to-moderate anemia, which affects both work capacity and productivity [16, 18]. Reducing anemia may also contribute to reducing gender wage gaps and help empower women economically [38]. In pregnant women, anemia can lead to increased risk of premature birth, low birth weight, permanent reductions in children’s cognitive capacity and maternal mortality affecting the next generation’s health across their life course [16, 19, 35].

Despite large-scale government programs to reduce anemia, prevalence has changed little over 15 years, with some improvements among pregnant and lactating women [17]. Although the aforementioned national distribution programs increase supply, actual IFA use in India remains low. According to the most recent National Family Health Survey, which only measures IFA use among pregnant women, 91% of pregnant women in India reported that they received IFA during their last pregnancy but only 37% consumed them for more than 100 days during pregnancy, suggesting potential demand side problem [18]. Adolescents in schools are also fairly well covered compared to out of school non-pregnant women. In a 2016 study in rural India, almost half of adolescents in schools reported consuming IFA tablets regularly, and a little over half were consuming occasionally or rarely [30]. While these programs have increased IFA supply to adolescents and pregnant women, increasing supply in subpopulations without demand-side efforts is unlikely to substantially increase uptake. All of this raises the question as to how IFA uptake can be accelerated more effectively.

Considering different levels of the socio-ecological model, individual knowledge, attitudes, and beliefs about IFA and anemia has been highlighted as fundamental to IFA use [8, 26]. At the interpersonal level, a growing body of literature also points to social norms as a barrier to IFA uptake and particularly perceptions that most people do not take IFA [26, 42]. Social norms are informal rules of behavior considered acceptable in a group or society. Prior research on IFA use also points to the influence that mothers-in-law and husbands have on women’s likelihood to take IFA [2]. Moreover, recent research in India shows that inequitable gender norms, a sub-set of social norms that describe how people of a particular gender are expected to behave, in a given social context [3], may indirectly affect a woman’s ability or desire to access IFA [8, 11]. While we know social norms, including gender norms, are an important construct, we know little about the types of norms that matter, and if changing norms are likely to change IFA behavior.

At the community and policy level, a 2015 study on scaling up successful IFA interventions in India reported that stakeholder buy in and including anemia in the larger socio-epidemiological and politico-developmental context is critical [22]. On the supply side, a 2018 study in Bihar identified several community and statewide IFA bottlenecks including lack of IFA supply forecasting, lack of buffer stock at local levels, and inconsistent training on IFA counseling and distribution across different frontline workers [43].

To design effective interventions to increase IFA use, it is critical to examine barriers at every level of the socio-ecological model together and to understand how they interact in a particular context. However, existing research focuses on IFA supply, IFA use, and IFA adherence among pregnant women and adolescents, and neglects non-pregnant women of reproductive age.

To our knowledge, no studies have qualitatively examined all levels and behaviors related to taking IFA that include all women of reproductive age and their social networks. This paper seeks to answer the following research questions:

1. What are the multi-level barriers and facilitators to IFA use among all women of reproductive age in Odisha, India?
2. What are the most promising areas to intervene to increase IFA use in this population?

We take a theory driven approach to examine the multilevel factors and social norms that affect IFA use. We report the results of the first phase of a project, (The RANI Project, Reducing Anemia through Normative Innovations) [37]. We will subsequently develop a social norms-based intervention and test whether this intervention can increase IFA use in the treatment arm compared to the control arm via a cluster-randomized trial in rural Odisha, India [45].

## Theoretical framework

To address the multi-level factors that may affect IFA use, we use the socio-ecological model to frame our work [13]. The socio-ecological model posits that factors at various levels uniquely and jointly contribute to health and well-being. Given that prior research highlights the role of social norms on IFA use and the need to reach beyond individual factors [8, 36], we explore *how* social norms affect behavior. Therefore, the Theory of Normative Social Behavior (TNSB) also underpins this research. The TNSB can help elucidate *when*, *how and which* norms affect health behaviors [32]. Social norms are communicated and negotiated through social interactions and therefore operate at the interpersonal or community level [9]. Due to our natural human desire to connect with others, the power of normative social influence can directly impact behavior [4, 39].

The TNSB states that perceived descriptive and injunctive norms may impact behavioral intention, which may in turn impact ones’ behavior. Perceived descriptive norms refer to individuals’ beliefs about what other people do and how often they do it (e.g., most women in my community take IFA). Perceived injunctive norms are what individuals believe that others expect them to do (e.g., pregnant women should take IFA daily so they have a healthy baby). Perceived descriptive norms are thought to influence behavior because of people’s desire to do the right thing or the thing that they believe most people are doing. Perceived injunctive norms are thought to influence behavior because of individual’s motivations for connection with others [10]. At the social level, collective norms, reflects community-level attitudes or practices, and are often measured as the aggregate of attitudes or behaviors in a specific area. Collective norms illustrate how likely someone is to come into contact with an attitude or behavior in their community and is an indication of how accepted that attitude or behavior may be [33]. According to the TNSB, it is also important to assess one’s referent groups or social networks [23]. Informed by the social ecological model and the TNSB, we explored potential modifiable attitudes, beliefs, norms, and behaviors within the whole context of women’s lives.

## Methods

### Study setting

Odisha is a state on India’s Eastern Central Coast along the Bay of Bengal. 83% of households in Odisha reside in rural areas and 64% of women and 82% of men are literate. The vast majority of household heads are Hindu (94%) and 23% of households belong to a specific tribal culture [7]. The total fertility rate in Odisha is 2.1 children per woman. About half (51%) of women in Odisha have anemia, with a higher rate for women from a tribal culture and those with no schooling [18].

This study was conducted in the Angul district, one of the 30 districts in Odisha, which has 1930 villages and a total population of 12,73,821 [7]. Angul lies approximately 130 km northwest from the capital of Odisha, Bhubaneswar. While some parts of Angul are known for mining and industrial activities, it is primarily a rural area where men often take farming or day labor jobs. Women often work in the home, in subsistence farming, and/or make bidi, homemade cigarettes, to sell in the market. Most households are co-living meaning that the husband, wife, children, and the husband’s parents, all live and eat together. In Angul, pregnant women obtain IFA from various types of frontline health workers: Auxiliary Nurse Midwives (ANM), Anganwadi workers (AWW) and Accredited Social Health Activists (ASHA). The ANM has the most education and is based at a health-center, the AWW works solely in her village and focuses on provision of food supplements to children, adolescent girls, and lactating women, and the ASHA also works in her village and focuses on maternal and child health, including immunizations, IFA supplements, and institutional-based deliveries. AWW & ANM are salaried workers while ASHA receive an incentive based honorarium. Both pregnant and non-pregnant women can go to their nearest health center to test for anemia and if they are diagnosed with anemia, obtain free IFA at the clinic dispensary set-up by the government.

### Data collection modalities

We collected three types of qualitative data including key informant interviews (KIIs), focus group discussions (FGDs) and structured observations. We stratified FGDs by village, gender, and age. We chose a homogeneous sampling method to create a comfortable environment that facilitated group discussion and to be able to describe subgroups in more depth [29]. We also conducted structured observation in each of the villages in several locations to examine the environment where women are obtaining IFA.

### Instrument development

We developed the interview, focus group, and structured observation guides for this study. They were based on the Theory of Normative Social Behavior, a literature review, and feedback from pilot testing in Odisha, India (See appendices A, B and C for all three guides). We conducted three pilot interviews with key informants and three FGDs from nearby villages to ensure participants understood the questions well and they aligned with the local manner of speech. We transcribed four pilot interviews to conduct additional analysis and then modified guides to improve the clarity in questions. Interview guides covered general questions about what women do on a typical day, their concerns and aspirations, and barriers to and facilitators of IFA use. To explore women’s social norms in a more approachable format, we used vignettes-- short stories about theoretical characters that also live in a rural village in Angul, India [14]. Vignettes can also help identify what if any social sanctions exist, as well as to test emerging hypotheses about the existence of a social norm [6].

### Sampling

We used a random sampling procedure to select FGD participants. Researchers enumerated each household in all four villages. Based on approximately ten women needed for each FGD, against a sample that included the entire village, we used a proportional skip pattern starting with a randomly selected participant to identify households to select each succeeding participant for each sub-group. We used purposive sampling to choose key informants who included frontline health workers, teachers, natural healers, medical doctors, and self-help group leaders [28]. Local frontline health workers helped to identify the most knowledgeable key informants from each village. All participants were asked to participate in person. We observed one pharmacy, health center, and village health and nutrition day from each of the four villages. These observations allowed us to examine availability of IFA or anemia related health information in the pharmacies and health centers.

### Data collection

Between March – May 2018, we collected formative research data from two blocks, (district sub-divisions), Kishorenagar and Athamalik, in four villages in Angul, Odisha. We conducted 16 focus groups with women of reproductive age, mothers-in-law, adolescent girls, and husbands (*n* = 124). We also conducted 25 key informant interviews with frontline health workers, medical doctors, natural healers, teachers, and self-help group leaders. KIIs were held in the participants workplace or home and FGDs were held in community meeting spots and health centers. Additionally, we conducted structured observations of 17 different locations (health centers, medicine stores/kiosks/pharmacies, self-help groups and village health and nutrition days) across the four villages (see Table 1).

**Table 1:** Data collection modalities

Focus groups		Key informant interviews		Structured observations	
Participant category	Number conducted	Participant category	Number conducted	Participant category	Number conducted
Women of reproductive age	4	Frontline Health Workers (ASHAs, AWWs, ANMs)	12	Local Markets	4
Mothers-in-law	4	Self-Help Group Leaders	4	Primary Health Centers	4
Adolescent girls	4	Medical Doctors	4	Pharmacies/Kiosks	4
Husbands	4	Teachers	3	Village Health and Nutrition Day	1
		Natural Healers	2	Self-Help Groups	4
**Total**	**16**	**Total**	**25**	**Total**	**17**

Our study team conducted a training in Bhubaneswar, Odisha that included mock interviews, reflexivity exercises, and ethics on human subject’s research. The reflexivity exercise helped us to acknowledge different experiences and opinions in an effort to reduce individual biases. The process of writing and sharing created a greater awareness of how our different backgrounds may affect how we ask interview questions or how we conduct the analysis [25]. The trained research investigators conducted all of the interviews face-to-face in Odiya and we matched interviewer and interviewee gender. We selected four rural communities for our study, to ensure that we had a heterogeneous mix of caste and tribe. For each focus group, one member of the research staff observed the group and took field notes and researchers wrote field notes following each KII. All KIIs and FGDs were audio recorded and our research partners transcribed all interviews in Odiya then translated them into English for analysis. Another Odiya speaking translator conducted an additional quality check after initial translation to ensure accuracy. We also asked participants to complete a demographic questionnaire. (see Table 2 with demographic information) and research investigators verbally explained the study goals and obtained written informed consent before beginning the interview.

**Table 2:** Demographic Information

	Women 15–35	Males	Mothers-in-law	Key Informants
	(*n* = 64)	(*n* = 30)	(*n* = 30)	(*n* = 24)
**Age**	***M (SD)***	***M (SD)***	***M (SD)***	***M (SD)***
	22.14 (5.94)	32.23 (6.10)	54.77 (6.03)	40.63 (12.02)
	**n (%)**	**n (%)**	**n (%)**	**n (%)**
**School**				
None	4 (6.3)	0 (0)	14 (46.7)	2 (8.3)
Up to Primary	5 (7.8)	10 (33.4)	11 (36.7)	0 (0)
Up to Secondary	13 (20.3)	7 (23.3)	4 (13.4)	4 (16.6)
Up to High Secondary	33 (51.6)	7 (23.3)	1 (3.3)	9 (37.5)
Up to Tertiary	9 (14.1)	6 (20.0)	0 (0)	4 (18.6)
**Married**	32 (50)	26 (86.7)	29 (96.7)	22 (91.7)
**Religion-Hindu**	64 (100)	30 (100)	30 (100)	24 (100)
**Caste**				
Scheduled Caste/Tribe	36 (56.3)	18 (60)	16 (53.4)	6 (25.0)
Other Backward Caste	22 (34.4)	10 (33.3)	13 (43.3)	14 (58.3)
Other Caste	6 (9.4)	2 (6.7)	1 (3.3)	4 (16.7)
**Children**				
None	34 (53.1)	7 (23.3)	0 (0)	3 (12.5)
One or two	23 (35.9)	15 (50.0)	7 (23.3)	17 (70.8)
Three	4 (6.3)	6 (20.0)	13 (43.3)	2 (8.3)
Four or more	3 (4.7)	2 (6.6)	10 (33.3)	2 (8.3)
**Ever taken IFA**	52 (81.3)	1 (3.3)	7 (23.3)	17 (70.8)
**Currently taking IFA**	6 (9.4)	0 (0)	0 (00)	1 (4.2)
**Diagnoses of anemia ever**	15 (23.4)	1 (3.3)	5 (16.7)	9 (37.5)
**Currently anemic**	4 (6.3)	0 (0)	1 (3.3)	1 (4.2)

### Analysis

We created a codebook draft using applied thematic analysis to characterize the relevant themes. To determine when we reached theoretical saturation, we used an iterative approach to data collection and analysis, whereby analysis and data collection happened concurrently [15]. We independently reviewed transcripts to identify initial themes and to develop a codebook. We used both inductive and deductive coding in the initial round of coding. We used specific a priori codes to identify text related to attitudes, beliefs, and norms related to IFA, and we added additional codes when new themes emerged during coding. We uploaded transcripts and the codebook into NVivo v.12 qualitative software for analysis [27]. Two researchers from The George Washington University and two researchers from our partner organizations from India, IPE Global Limited, and DCOR Consulting coded the transcripts. Our backgrounds are varied and range from a global women’s health researcher to a project manager who works to implement health interventions on the ground in Odisha. To ensure consistency across coders, we held weekly online meetings and the lead author reviewed several coded transcripts from each researcher. All coders met regularly over the course of analysis to discuss codes, review memos, reconcile discrepancies, and compare emerging themes.

To increase the validity of our data and to co-design the RANI study intervention, we presented our initial results to leaders who work in the anemia and women’s health field in Bhubaneswar, Odisha, India. We asked the group how our findings aligned with their experiences; they examined the rigor of the analytic process, and probed for potential biases thus increasing validity and encouraging transparency in our first round of analysis. This study was approved by Institutional Review Boards at the George Washington University and the Institutional Ethics Committee at DCOR Consulting.

## Results

**Fig. 1:**
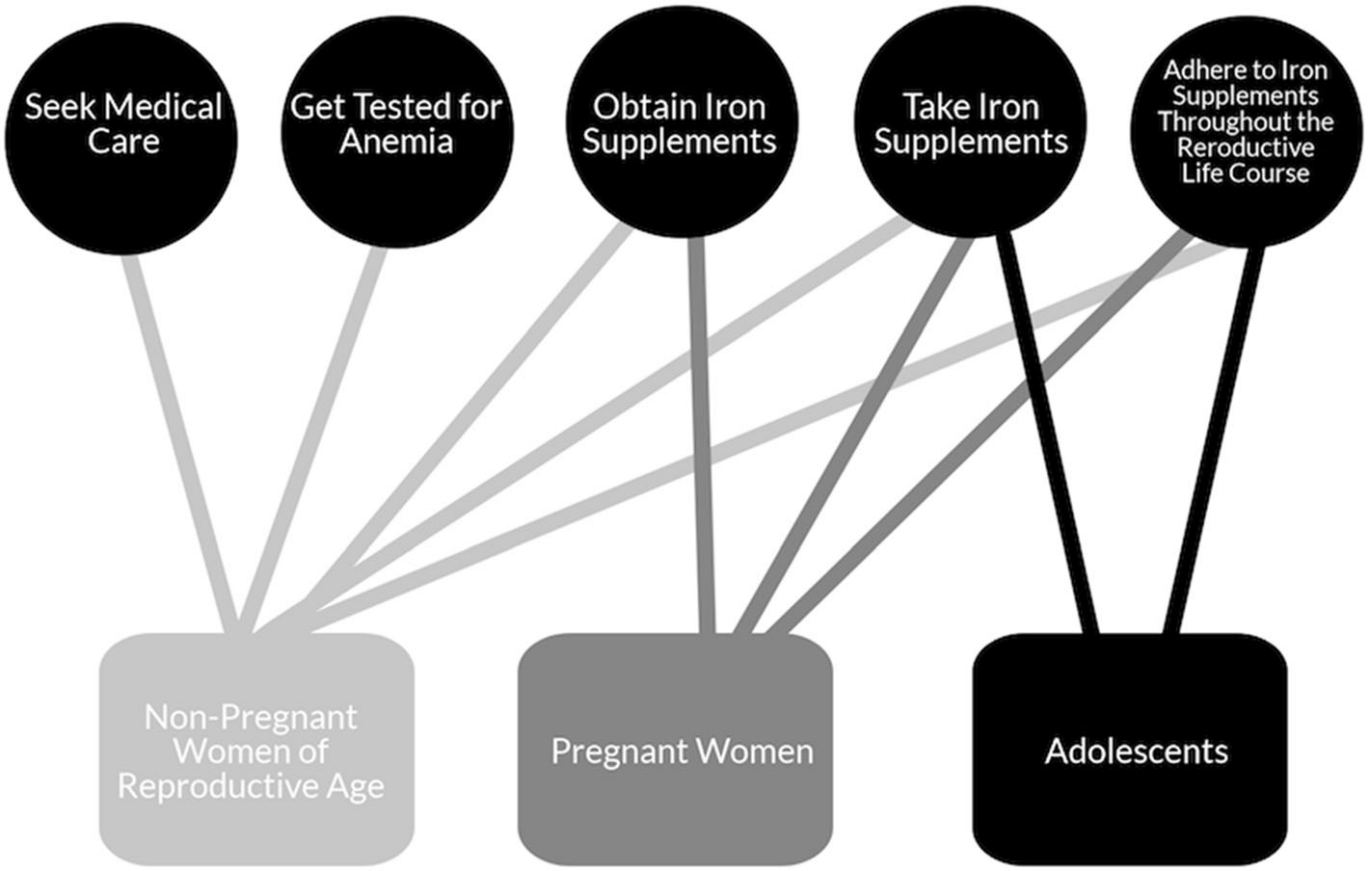
Behaviors by reproductive life course stage

A description of the sample included in our study is shown in Table 2. Based on our analysis, we quickly realized that participants were primarily referring to pregnant women and adolescents taking IFA (despite vignettes that asked about both pregnant and non-pregnant women taking IFA) and that barriers are quite different for these sub-groups of women. Therefore, we designed a theoretical model (see Fig. 1) to describe the spectrum of behaviors related to IFA for all women of reproductive age, including seeking medical care, getting tested for anemia, obtaining IFA, taking IFA, and adhering to IFA throughout the reproductive health lifespan. We found that this kind of behavioral specificity is largely ignored in the literature (which primarily focuses on IFA supply and adherence) and we wanted to examine each separately to understand where on this behavioral spectrum our target audience lies to design an intervention accordingly. It is important to note that not all behaviors are necessary to adhere to IFA throughout a woman’s reproductive life course (adolescents, pre and post pregnancy, and pregnancy). Specifically, adolescents can obtain IFA in school and pregnant women can get IFA from front line health workers. However, for non-pregnant women, these behaviors may be critical first steps to IFA use.

Several themes in the data describe why IFA use is low in this population:

(1) We found that participants knew that iron supplements prevent anemia but 2) grossly underestimated anemia prevalence and risk in their community which contributes to almost non-existent IFA use among non-pregnant women 3) The belief that taking too many IFA during pregnancy will “make your baby big” results in injunctive norms (especially from mothers-in-law) not to take too many 5) Unequal gender norms are an upstream barrier for non-pregnant women to prioritize their health to obtain IFA.

### Basic knowledge and mixed attitudes and beliefs about taking IFA during pregnancy exist

Most participants had some basic knowledge about IFA, what it is and that it “cures” anemia. Participants were well aware of anemia and referred it to as “lack of blood in the body.” A frontline health worker said, “If there is blood deficiency, anemia attacks.” There was some confusion around how one gets “lack of blood.” For example, some people believed that women get anemia from “not taking rest” or “heavy workload” while others knew that the main cause was “not consuming proper diet.” A natural healer said, “They have lack of blood because food is not right. They only eat salt and watered rice.” Most participants understood the near-term symptoms of severe anemia (primarily fatigue) but had limited understanding of long-term health consequences.

Most focus group participants knew what IFA (referred to as “iron batika”) was when the moderator held up the IFA pack and knew that they can prevent/cure “lack of blood in the body.” A woman from a focus group said, “We aren’t able to have proper food which creates blood in our body. These tablets (IFA) help in creating blood.”

Attitudes about the benefits of IFA use were predominantly positive. Almost everyone felt that IFA helps to keep people healthy and promotes a healthy pregnancy. A midwife said, “If we give fertilizers to plants, then, the plants will grow. They will stay fit and fine. Similarly, tablets (IFA) are fertilizers for human beings.” However, participants reported that they do not consistently take IFA because of “forgetfulness,” lack of “time,” and “side effects.” While the majority of people had positive attitudes towards IFA, two women of reproductive age noted some negative attitudes, “They fear taking those tablets. Women in the village think that they are bad tablets so they take them and throw them away.” Most negative perceptions stemmed from beliefs about side effects that outweigh the positive benefits of IFA.

The most salient negative perception was the belief that IFA will “make your baby big.” Every focus group and interview mentioned that if women take too many IFA tablets during pregnancy, they will have a “big baby,” which may mean a difficult labor or even a cesarean section, which would be financially costly. A mother-in-law said, “They fear IFA as some ladies tell them that the child will grow big in your womb.” Some beliefs are in competition with each other because most participants believe that IFA promotes a healthy pregnancy but taking too many will cause a big baby. As one man from a focus group said, “They think that the child will be born healthy [if you take IFA] but they also fear that the child will grow big in your womb.” A couple of frontline health workers stated that this belief does not exist in her community anymore but this was an exception, “In the tribal regions towards Koraput, people believe that iron tablets may increase the size of the child. Here they used to think so but now there is nothing such in our locality.” Other perceived negative side effects include “bad taste,” “bad smell,” “black stool,” and “bad breath.” A nurse midwife said, “Some people vomit due to the fishy odor, and we already explained that there will be black stool, nothing to fear about.”

### Perceptions of low prevalence of anemia in the community and non-existent IFA use among non-pregnant women make anemia and IFA a low priority

Despite endemic rates of anemia, perceptions about risk and prevalence are extremely low. Most participants were not concerned about anemia in their village. An AWW said, “In our village, in a year, 2-3-4 women have it [anemia]...mainly pregnant women.” Table 2 demographic data shows that most participants in our sample have not been diagnosed with anemia and are not currently taking IFA. A medical doctor reiterated this statement, “They understand that they have shortage of blood but they don’t take it seriously.” Generally, people believed that pregnant women are at the greatest risk for anemia and that non-pregnant women’s risk for anemia was only among the poorest who cannot afford nutritious food.

Participants reported that only adolescent girls, lactating women, and pregnant women (intermittently to prevent a big baby) are taking IFA. A man from a focus group said, “That [IFA] is only given to pregnant women. They say that the pregnant women lack blood and the normal [non-pregnant] people don’t take it.” A doctor said, “In the schools, they give it [IFA] to children with the mid-day meals and our ASHA and ANM sister distributes it to pregnant women.” The medical doctors report that their patients often do not come back to the medical center when asked to and rarely complete their prescription. A few participants reported that, “educated girls take the iron tablets to some extent.” It is also important to note that only 9.4% of women in our sample ages 15–35 years old reported that they are currently taking IFA (*n* = 6 out of 64 total WRA in our sample). Therefore, descriptive norms that only adolescents and pregnant women are taking IFA appear to be in line with collective norms (an aggregate of existing behaviors) in this study population. Furthermore, no participants, including key stakeholders, mentioned the National Indian Guidelines, which state that all women of reproductive age should be taking IFA [17].

### Social influence not to take too many IFA during pregnancy exists

Social influence not to take IFA is stronger than injunctive norms to take them. Most mothers-in-law did not take IFA when they were pregnant and do not feel that it is a priority. A mother-in-law said, “We never had those tablets. We had no idea that we are pregnant until the 5th month but women of today know everything from the 1st month and run to the hospital.” Additionally, some participants believed that mothers-in-law are perpetuating the big baby myth. As one midwife said, "Their in-laws scolded me by accusing me that the iron tablet made the baby overweight inside the womb for which caesarian section was necessary. You may hear them [mothers-in-law] say, “You don’t take more! She has told you take two tablets, you take only one.” Others were in favor of their daughters-in-law taking them for the health of the baby.

Our data provide little evidence of a strong injunctive norm in support of IFA use. We did not find any examples of participants stating there would be social consequences or social sanctions if they did not take IFA. Most husbands were supportive of their wives taking IFA during pregnancy but not overly concerned with them. One doctor said, “I have never seen men asking their wives to stop taking the tablets. It is either the women themselves who don’t take or the mothers-in-law ask them not to take.”

Another factor that may affect IFA use is that current state and national policies around who should take IFA are inconsistent. While the government has recommended preventive IFA for adolescents and pregnant and lactating women for decades, only since 2013, has the government recommended that all women of reproductive age take IFA. However, what is written in national policies does not appear to translate to this population [17, 24]. As one man from a focus group said, “The adolescent girls should take it. And whoever is suffering from fever in the village and have deficiency of blood, he or she will take this tablet.” One woman from a focus group reiterated that IFA are not part of a preventive health routine, “If physically sound and everything is normal then why should she take? She will never find any interest to take the tablets.”

### Unequal gender norms are an upstream, persistent barrier to IFA access

Gender norms regarding self-care are a barrier to accessing IFA. Women face strong expectations (i.e. injunctive norms) to ignore their own wellbeing to complete their workload and to serve their family. A doctor said, “They [women] don’t give themselves importance. They immediately go to the hospital and bring medicines for their husbands and children and they sacrifice their own health.” We found that women’s health and wellbeing ranks as last priority after her children, husband, and in-laws.

Expectations that women should work taking care of their family all day long may make it difficult to find the time to visit a health worker for IFA. It may not be socially acceptable to prioritize preventive health beyond pregnancy, which is focused more on the child’s health than the woman’s health. One woman said, “We have to work all the time, go to the mountains and fields, have children to take care of. We get no time to rest. We work in unhappiness and eat in pain.” However, some participants thought that women should also take care of themselves if only to be able to take better care of their families. As one natural healer said, “They [women] should take care of themselves first because if you are ill, you can’t take care of your child.” Men were aware that women were doing most of the housework while they did most of the manual labor outside of the home and they wanted their wives to be healthy (and/or the baby to be healthy). Mothers-in-law reported that daughters-in-law have it “easier” today than in the past so their expectations of their daughters-in-law were often more demanding than the husbands’ expectations of their wives. While participants and frontline health workers reported that IFA access is not an issue, more upstream barriers like unequal gender norms may make it difficult for women to access IFA representing a discrepancy between perceived and real access.

Overall, participants stated that IFA supplements are free and available from frontline health workers for pregnant and lactating women and that adolescents get them in school. As one natural healer said, “There has never been any kind of problem to get those tablets.” Adolescents take them weekly in school and medical doctors prescribe them with no cost after an anemia diagnosis." Some medical doctors also reported that ASHAs dispense IFA but do not provide enough information on why women need to take them, when they should take them, and for how long. On the other hand, some participants reported that ASHAs educate women about when and why to take IFA. As one mother in law from a focus group said, “They [ASHAs] explain how to take, when to take and how many days to take without fail.” Generally, people trust ASHA’s, ANMs, Anganwadi workers, and medical doctors. There was some discussion that IFA from the government was lower quality and that “educated families don’t take the free tablets. They take the tablets that they buy from medicine stores.” In general, people trusted the government to look out for their best interest. One medical doctor reported that they do not always have enough IFA in stock and sometimes run out before the next shipment arrives.

Figure 1 Shows where on the behavioral spectrum non-pregnant women, pregnant women, and adolescents currently fall. The figure shows that given that adolescents get IFA in schools they simply have to take it and continue to take it. Pregnant women can get it from front line health workers but they may have to seek them out or visit a village health and nutrition day. Among non-pregnant women, until current guidelines are disseminated and practiced among frontline health workers, they may have to start from earlier behaviors, including seeking medical care and getting tested for anemia to obtain tablets

### Structured observation results

While all pharmacies, health centers, and village health and nutrition days had IFA stock (free from the government and at cost at the private medicine stores), during the structured observation, some of the health center or pharmacy staff reported that they sometimes have stock outs. Monthly medicines were distributed from the district level distribution points. Additionally, pharmacists/medicine store-owners only sold IFA to people with a prescription from their medical doctor. At village health and nutrition days, frontline health workers provided IFA after testing for anemia. Research investigators observed that health staff provided only 30 tablets at a time, meaning that women would have to return monthly to obtain more. There were no pamphlets to explain why and when to take IFA or to address misconceptions around IFA in any of the health centers. Anemia-related posters for adolescent girls were only on the walls in two out of four health centers. Lastly, according to the person in charge of one medicine store, primarily pregnant women, children, and older men and women suffering from anemia were advised to take IFA.

## Discussion

We identified a spectrum of behaviors that women may need to perform to access, use, and adhere to IFA. Adolescents, pregnant, and non-pregnant women have different sets of behaviors to adhere to IFA due to different services available to them. We found that most participants had some knowledge about IFA and attitudes and beliefs were primarily positive but the belief that taking too many IFA during pregnancy will cause a “big baby” trumped positive outcome expectations. Additionally, risk perception of getting anemia is low as most women did not believe that many women in their community have it. Women’s social network’s attitudes and beliefs around IFA affect their use. Descriptive norms or perceptions around who is taking IFA state that only pregnant women and adolescents are taking IFA. Finally, gender norms and an evidence-to-practice gap among front line health workers may make it difficult for non-pregnant women to access IFA.

Our results are similar to other studies in India with some notable differences. From an individual level perspective, other studies have found that side effects, bad small and taste, forgetfulness, and limited information about IFA from frontline health workers hinder use [20, 31, 42]. Prior studies also show that women believe that taking IFA during pregnancy may lead to a large baby, making labor more challenging [8, 26]. Recent studies have similarly found that health workers are trusted and effective at distributing IFA [42]. A meta-synthesis of qualitative research on the social determinants of iron supplementation among women of reproductive age conducted in 17 countries identified social norms as one of the primary factors limiting uptake of IFA [26]. Two studies examining IFA use among pregnant women also found that unequal gender norms and lack of household decision-making is an impediment to IFA use [8, 11]. While IFA were in stock during our one-day observations, providers reported that stock outs were frequent [43]. also reported stock outs [43] but other studies have similarly found that demand issues play a larger role than supply issues [42].

Our data illustrate that attitudes about taking IFA during pregnancy are primarily positive (despite intermittent use to prevent big baby). However, we also found that risk perceptions around anemia are low. Therefore, positive attitudes may not be enough to adhere to IFA throughout pregnancy. Even if one believes that IFA may prevent or cure “lack of blood,” if one does not believe that it is a serious health problem, positive attitudes may not be enough to change behavior. It is important to note that these low risk perceptions may also stem from the fact that participants were often referring to severe anemia not mild or moderate anemia as the focus group moderator did not differentiate between the three levels.

**Fig. 2:**
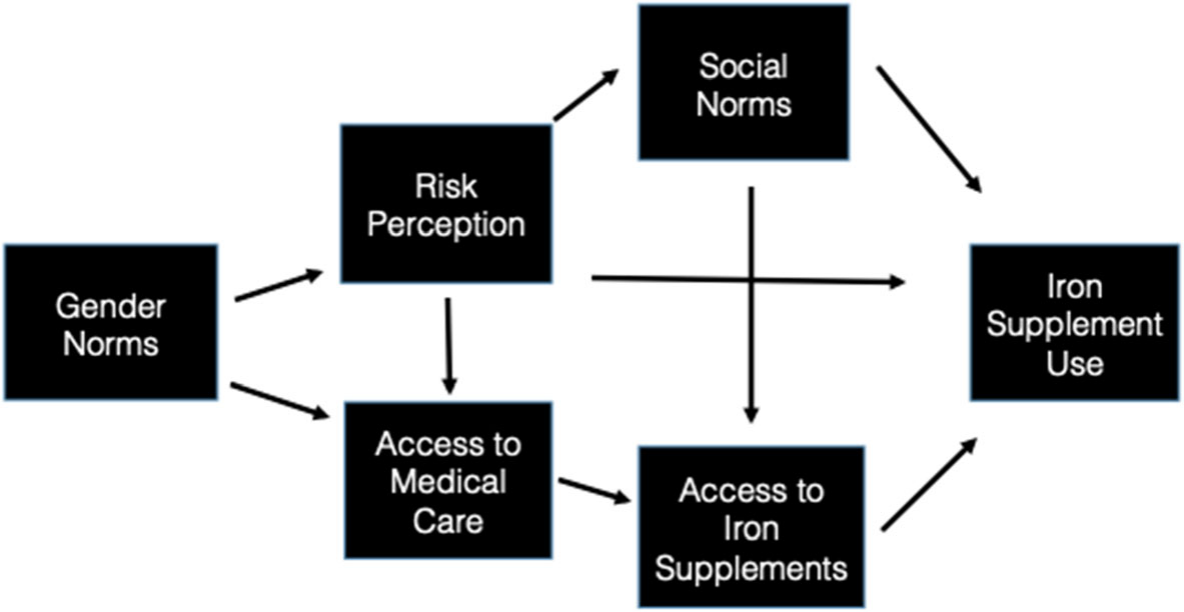
How factors interact to influence behavior

Our data point to a few ways in which different types of social and gender norms may interact to affect IFA use (see Fig. 2). Unequal gender norms that normalize fatigue (the most common symptom of anemia) among women may be negatively affecting women’s perception that they may be at risk for anemia. Similarly, women’s preventive health is not prioritized in the family, thus reducing access to preventive care. Additionally, perceptions that non-pregnant women are not at risk for anemia affects descriptive norms that only pregnant women and adolescents are taking IFA. These norms also trickle down to frontline health workers who only distribute IFA to pregnant and lactating women.

The Indian government IFA guidelines are not translating to practice on the ground. To close this policy-to-practice gap, evidence-based implementation science strategies from low and middle-income countries should be used [34]. Frontline health workers may be missing a critical opportunity to improve community member’s knowledge, attitudes, and beliefs about IFA. Simply distributing IFA will not change embedded norms and opinions of important reference groups like mothers-in-law. Additionally, frontline health workers activities only include distributing IFA to pregnant and lactating women, thereby missing the majority of women who have anemia (non-pregnant women of reproductive age). Therefore, if non-pregnant women want to take IFA, they need to go to a health center to be tested for anemia. Furthermore, frontline health workers do not receive incentives to follow up with pregnant women to ensure adherence beyond distribution.

### Intervening factors

**Fig. 3:**
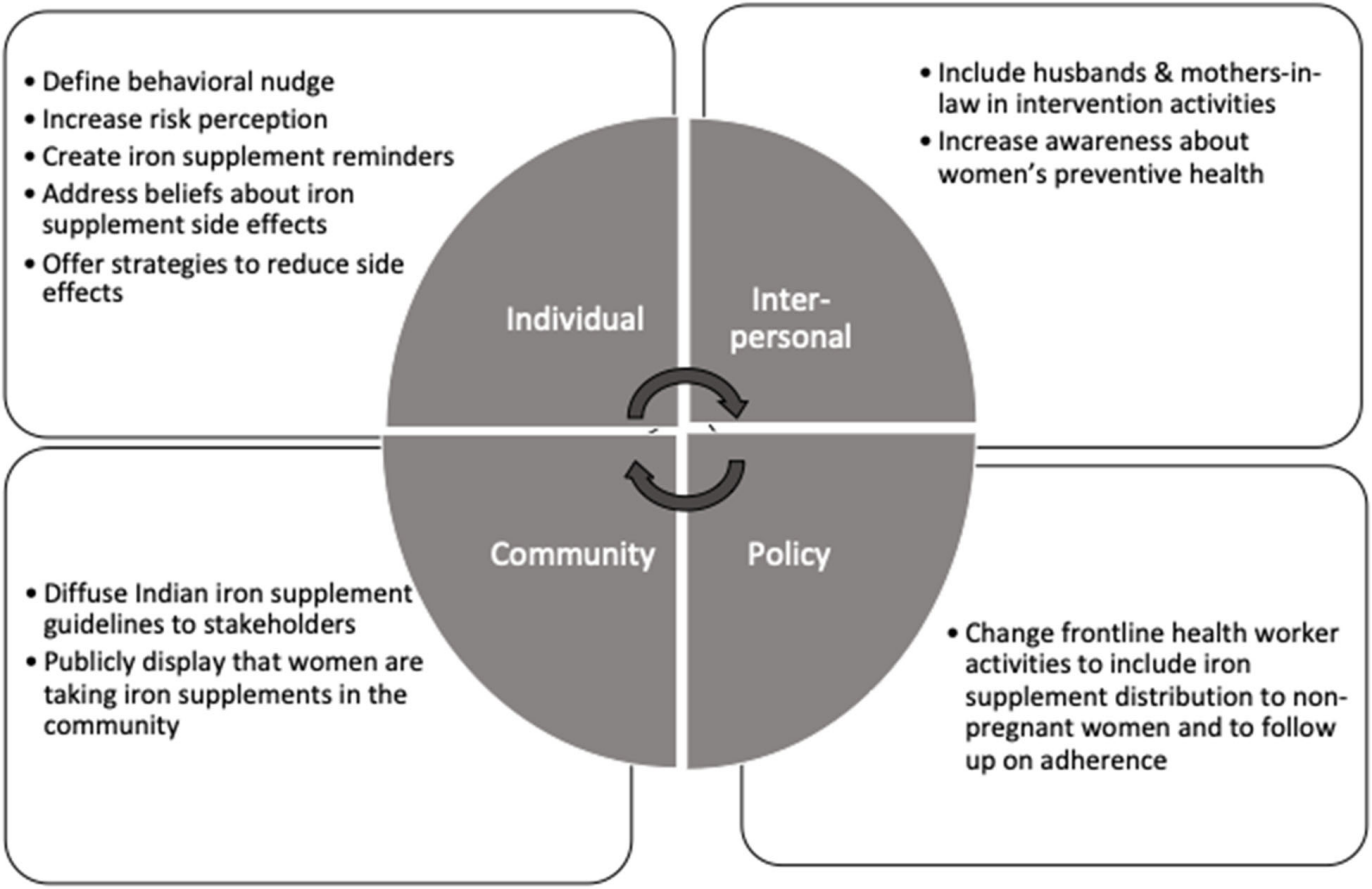
Suggested intervening factors

Figure 3 suggests areas to intervene at multiple levels of the socio-ecological level based on our study findings. Our research suggests that interventions should segment women of reproductive age according to readiness to change, which is in turn mostly dictated by reproductive life course stage, as non-pregnant women are not aware that they should be taking IFA regularly. While interventions for pregnant women should focus on knowledge, attitudes, and beliefs to take and adhere to IFA, interventions for non-pregnant women may also want to address social and gender norms that hinder seeking preventive care at all. A broader definition of access beyond supply and cost needs to take into account more upstream barriers like gender norms that may affect true access (e.g., ability to leave the house).

Our findings that unequal gender norms make it difficult to prioritize women’s health means that messaging may need to begin to raise awareness about women’s preventive health prior to moving onto taking IFA. On the other hand, gender norms that connect womanhood with hard work on behalf of the family could also be a facilitator of, rather than a barrier to, IFA use. After all, if accessing IFA is relatively easy, and if taking IFA gives women the energy they need to perform their roles, then interventions could frame IFA as a valuable investment not just to the woman herself but to the family as a whole.

Clearly, knowledge about the prevalence and risks of anemia is critical as risk perception may be connected to gender norms. If women believe that their role as women is to bear fatigue and not to worry about prevention efforts, increasing risk perception is an important step. Furthermore, among pregnant women, debunking misperceptions about “big baby” is critical. Women are hearing mixed messages from frontline health workers and from their mothers-in-law. Intervening factors for pregnant women should focus on correcting misperceptions to encourage women to take them, and reminders and tips to increase adherence. Real side effects are also important to consider. To counter these, women need to be armed with information about when to take them (e.g., with food) and that the benefits outweigh the costs. Due to the low status of women, focusing on benefits to the unborn child or increased energy to provide for the family may be more effective than communicating health benefits for the woman. However, to get at the root of one of the social determinants of this disease, gender norms need to be addressed [12]. Moreover, mothers-in-law and husbands are key players in the household and all intervention activities should also target them. Including these reference groups may increase injunctive norms so that women feel pressure from their reference groups to take IFA.

Additionally, we found that frontline health workers lack in-depth knowledge about anemia and IFA. Educating them about the benefits of IFA consumption and correcting their misperceptions could be an efficient way to communicate. An even more upstream approach may be to change ASHA/AWW incentive structure so that they are incentivized for adherence not just distribution of IFA. Finally, statewide consistency and messaging around who should be taking IFA would increase use among non-pregnant WRA.

### Limitations

This work has several limitations that may impact the interpretation of the results. One is external validity - our study is limited to four rural communities in Angul, Odisha so it may not be representative of rural India as a whole, or urban areas of Angul. Researcher bias is also a threat to validity during the analysis process. Our personal and professional experiences and beliefs can bias analysis and reporting. This may result in an over or under emphasis of certain themes. To mitigate this threat, we included a diverse range of coders from different fields, different genders, and different countries, the United States and India, to carry out investigator triangulation. All coders also kept regular memos reflecting on their backgrounds, prior research experience, and how this may impact their interpretation of the results. While we collected demographic information about caste and tribe, we did not stratify by caste. Including participants from different castes in the same FGD may have made it difficult for participants from a lower caste to feel comfortable to express their opinion. We did take this into account during the focus group recruitment and ensured that each focus group took place in a neutral location that was not affiliated with a specific caste. Furthermore, while we assessed IFA stock with structured observation, we only visited each distribution site once. A more accurate picture of supply side issues should take place over a longer period of time. To mitigate this limitation, we triangulated our observations with interviews with medical providers who reported past stock outs.

## Conclusion

Our results illustrate how barriers at different levels of the socio-ecological model interact to affect IFA use. We found that adolescents, pregnant, and non-pregnant women have unique barriers and therefore require tailored interventions. Women’s social networks disseminate misperceptions around IFA use and low perceptions about anemia risk and prevalence deprioritize the illness. Social norms around who is and who should be taking IFA and unequal gender norms make it difficult for non-pregnant women to access IFA. Finally, government guidelines are not reaching non-pregnant women elucidating an evidence to practice gap. Although intervening at multiple, upstream levels will be challenging, it may be necessary to truly increase IFA use to reduce anemia among all women of reproductive age in India.

## Data Availability

The data used and/or analyzed during the current study are available from the corresponding author upon request.

## Appendix A

### Focus Group Guide

Before Beginning the Interview:

- Welcome participant and introduce yourself

Hello, and thank you for speaking with me today. My name is ______ and I am a field researcher at DCOR. I am conducting a research study to understand the attitudes and beliefs of people in this community. Thank you so much for agreeing to participate and taking time out of your day.

Do you have any questions about the research and your participation before we start?

- Review key points, ethics and confidentiality policy:
  - • You will not gain any direct benefit from participating in this research; however, we hope our results will be used to reduce anemia in Odisha.
  - • We do not anticipate you will experience any risks but please feel free to not answer any questions that you are uncomfortable with or to stop the interview at anytime.
  - • The discussion in this interview is completely confidential
  - • Your responses will be kept confidential and your name will not be cited on any written materials coming out of this study.
  - • There are no right or wrong answers to the questions I am going to ask you. All experiences are important
  - • The interview is being tape recorded so that we can accurately capture what you’re saying, but no one besides the research team will have access to it.
  - • You are free not to answer any questions that make you feel uncomfortable or to stop the interview at any time
  - • We expect the interview to last about 1 h

#### Warm-up Questions

I’m new to this community, [name District], can you tell me a little bit about it? For example, what kind of food do people eat here? And what do people do to earn money?

### Women’s Role in the Community

What are some of the typical things that women in this community do throughout the day? (probe: things like cooking, cleaning, taking care of kids, working outside the home, spending time with her husband).

Do women in this community have enough energy or time to these things each day?

- Look after her kids?
- Work outside the home?
- Cook?
- Spend time with her husband?
- Have more intimate time with her husband?

How are men and women treated differently in this community?

For women’s life in general, are things getting better or worse?

- in her role as a mother?
- in her role as a wife?
- in her role as a daughter in law?

What are some of the concerns that women in this community have? (probe: concerns about health, money, their family, themselves, their future)

- Some people think that (woman’s name) should first take care of her husband and kids before she worries about her own health. Other people think that she should first take care of her own health.
  How do you think most women in this community feel about that? What about most men?
  In some families in Odisha, women eat after their husband, children and mother in law eat. In others, women eat at the same time. What do you think about that? Is this changing?

#### Anemia and Iron Folic Acid (IFA) Knowledge

(Show an IFA tablet and liquid IFA)

Can someone tell me what this is? (probe: What does it do?)

Have you ever heard of anemia?

Many people in this community may not use the word “anemia” but they may have other words or phrases to talk about anemia. Can you tell me what some of those phrases are?

Tell me a little bit about what happens when someone has anemia (use the word or phrase identified above instead of “anemia”). Tell me how this person feels or how this person acts when they have anemia.

What do you think causes anemia? (probe: Not eating enough iron rich foods?)

What do you think makes anemia go away? (Probe: Can IFA tablets help? How about changing your diet? What kind of foods might help it go away?).

#### Anemia Related Behavior

Ok, now we’re going to make up a character. She is a female aged 23 years old. What should we call her? (Ask for suggestions and decide on a name together). She just got married and is 3 months pregnant with her first child. Her doctor just told her she has anemia.

The doctor told her to take iron tablets.

- If (same woman’s name) wanted to get IFA tablets, where could she get them? How difficult is it to *get* IFA tablets?
  - ◦ Can you tell me what she likes about taking the tablets and what she doesn’t like? (PROBE by asking more likes and dislikes)
  - ◦ Apparently, some women stop taking those tablets. Can you tell me why they stop?
  - ◦ Can you tell me what good things may happen if someone takes the tablets?
  - ◦ What do you think would help her take the tablets? Do you think (woman’s name)‘s husband will support her to take the tablets? Not support her to take them? Or not say anything? (Ask for examples)
  - ◦ What about (woman’s name)‘s mother in law? Will she support her in taking the tablets? Why or why not?

#### IFA Norms

- Is Anemia is a problem in the community? If yes How much of a problem?
- In general, who is typically expected to take IFA tablets? (Probe: pregnant women, adolescent girls, non-pregnant women?)
- Please think about most women like (name) who live in this community. How many of them take IFA tablets? Some? Few? Most?
- Is there anyone in (woman’s name) family (or her husband’s family) that she can talk to about taking IFA tablets? How much do you think she’ll listen to what they have to say about IFA tablets?

#### Information Sources

Imagine another woman who is not pregnant but interested in learning more about her health. What should we call her? (Ask for suggestions and decide on a name together).

Where does she go for information about health related matters?

Where can she get information about pregnancy, anemia, and IFA tablets from?

- How easy is it for her to get information about pregnancy, anemia, and/or IFA tablets? What difficulties would come up when trying to get this information?
- When a woman gets IFA tablet, what kind of information does she get about them?
- Probe: Dose, when to take or why to take it?
- For those of you that participate in self-help groups, can you tell me what kind of things you discuss?

#### Closing

That is the end of the questions I have for you, but do you have anything else you’d like to add to the discussion? Any little stories about anemia or IFA use in this community?

As a reminder, please do not share anything we spoke about today with anyone outside of this group.

Any questions?

Thank you for your time.

## Appendix B

### Key Informant Interview Guide

Before Beginning the Interview:

- Welcome participant and introduce yourself

Hello, and thank you for speaking with me today. My name is ______ and I am a (your role) at ______. I am conducting a research study to understand the attitudes and beliefs that effect IFA tablet use in _____(selected county)____.

Thank you so much for agreeing to participate and taking time out of your day.

Do you have any questions about the research and your participation before we start?

- Review key points, ethics and confidentiality policy:
  - • You will not gain any direct benefit from participating in this research; however, we hope our results will be used to reduce anemia in Odisha.
  - • We do not anticipate you will experience any risks but please feel free to not answer any questions that you are uncomfortable with or to stop the interview at anytime.
  - • The discussion in this interview is completely confidential
  - • Your responses will be kept confidential and your name will not be cited on any written materials coming out of this study.
  - • There are no right or wrong answers to the questions I am going to ask you. All experiences are important
  - • The interview is being tape recorded so that we can accurately capture what you’re saying, but no one besides the research team will have access to it.
  - • You are free not to answer any questions that make you feel uncomfortable or to stop the interview at any time
  - • We expect the interview to last about 1 h

#### Warm-up Questions

Can you tell me about your role? I’m new to this community [name district], can you tell me a little bit about it? Like what do people do to earn money? What types of jobs are normal for women to have? How often do people move in and out of the town? What do people eat here?

#### Anemia in this Community

How common is anemia in Odisha? Is it a state priority? A country priority?

How does the diet in Odisha contribute to anemia? (probe: for example do people here eat iron rich foods like meat or green leafy vegetables?)

#### Knowledge about Anemia

Can you tell me a little bit about how big of a problem anemia is in this community? What is your understanding of some of the health risks of anemia?

How aware are residents in Odisha of the prevalence of anemia? How familiar are they with the health risks of anemia?

#### IFA Availability

Are you familiar with IFA tablets? How about IFA syrup?

Can you tell me who distributes the tablets and how? (probe: Are they private or government distributors?)

How widely dispensed are the IFA tablets? In your opinion, how easy is it for the average resident of [say village] to get IFA tablets?

Typically, how much do IFA tablets cost? Is this cost a lot to people in the community? How many tablets does a person get at a time?

If a woman wanted to get IFA tablets, where could she get them? How difficult is it to *get* IFA tablets?

#### IFA use

How difficult is it to *tak*e IFA tablets? (probe: what are some of the reasons that someone stops taking them?)

How difficult is it to *continue to take* IFA tablets regularly? How about taking them every day? probe: what are some of the reasons that someone stops taking them?)

Can women in Odisha overcome these barriers? What would help?

#### Prior Interventions

Which programs in this community try or have tried to reduce anemia or increase IFA tablet use? Can you describe these programs to me? How well liked were they? What worked well for the programs and what didn’t?

What, if at all, was the relationship between the other programs on anemia and IFA use and the self-help groups?

#### Barriers and Facilitators to IFA use

What do people in the community know about the benefits of taking IFA tablets regularly? (probe: do most people in this community know about the connection between IFA tablets and anemia?)

What do people think are the drawbacks of taking IFA tablets? (probe: what are some of the potential side effects of the tablets? How do they feel about these side effects?)

#### IFA Norms

Who is typically expected to take IFA tablets? (probe: men, all women, adolescents, only pregnant women? how regularly? When do they take them? Morning, after a meal, bedtime?)

Please think about most women who live in this community. How many of them take IFA tablets? Some? Few? Most? Are they motivated to take IFA? To what extent?

Please think about most men and children who live in this community. How many of them take IFA tablets? Some? Few? Most? How motivated to take IFA?

Is there any pressure to take IFA? (Probe: How much pressure exists to take IFA tablets? Where does this pressure come from? family, friends, health systems, etc.)

#### Role of Health Workers (Aganwadi worker, ANM and ASHA)

Can you tell us a little bit about the Aganwadi workers, the ANMs and the ASHA? What is their relationship with each other?

What is their relationship with the self-help groups?

#### Self Help Groups

Are there any SHGs in this village? What are they expected to do? (probe: what kinds of things do they discuss? What type of women join? Why do they join?)

What are the attitudes within the self-help groups? (probe: do people view it as helpful? In what ways do you think it is helpful?)

What is the SHGs relationship with other departments like? (Probe: how do they interact with the ministry of health or local NGO’s?)

#### ANC/Anemia Care System

What are some other places where women can find information about anemia or IFA tablets? (probe: how about antenatal care clinics when they’re pregnant?)

What are the common screening and treatment practices for anemia?

#### Healthcare access

Where can someone get information about health? (Probe: health clinic, from family members).

Where can someone receive information specifically about anemia, and IFA tablets? (probe: how often do women visit or speak with them?

How easy is it to get information about anemia, and/or IFA tablets? Does the amount of information that women get about anemia or IFA change during pregnancy?

Are traditional healers or doctors more commonly used? (Probe: why is one is better than the other?)

Are private or government run health clinics more commonly used? (Probe: why is one is better than the other? How about to get screened for anemia? How about to get IFA tablets?)

#### Closing

That is the end of the questions I have for you, but do you have anything else you’d like to add to the discussion? Any little stories about anemia or IFA use in this community?

Any questions?

Thank you for your time.

## Appendix C

### Structured Observation Check Lists

**Antenatal Care Health Centers & Kiosks/IFA dispensaries:**

- ◦ How are IFA tablets dispensed? Please describe the process.
- ◦ Describe the general conditions of the clinic/kiosk (cleanliness, crowding, wait-time, etc.).
- ◦ Are IFA tablets available at the clinic? What is the cost?
- ◦ How are IFA stocked?
- ◦ If possible to observe, how often are they provided to pregnant women and to non-pregnant women of reproductive age?
- ◦ Are there educational materials about anemia or IFA tablets in the clinic? Any other health related materials?

**Self-help Groups:**

- Where do the self-help groups meet? What type of area are they in? What is the composition of members (age/caste)
- Describe the type of information given to group participants
- Describe the activities conducted.
  - ◦ What is the nature of the activity?
  - ◦ Who participates?
  - ◦ Who leads the activity?
  - ◦ What information is exchanged during the activity? Is there anything related to anemia or IFA tablets? General health information?
- Describe any interactions related to anemia or IFA.
- How well functioning does the group appear? What is the overall sentiment from participants and leaders?

**Food markets & general community observation:**

- Describe all anemia or IFA related media content. Where were these found?
- Describe all health related content. (posters, advertisements, brochures, etc.)
- Describe the engagement in physical activity.
  - ◦ What types of physical activity occurs in daily life?
  - ◦ How often do women walk? What is the speed? How often do they sit versus stand?
- Describe the food that is purchased or eaten. What is the quantity? Variety? What types of vegetables? Does the food vary by season?
- Are people growing their own food in a garden or on a farm? If so, how much and what kinds?
- What type of labor (work) are people doing in the village? How physically active is it? Are both men and women participating?

## Abbreviations

IFA: Iron folic acid supplements
WRA: Women of reproductive age
RANI: Reducing Anemia through Normative Innovations
TNSB: Theory of Normative Social Behavior
ANM: Auxiliary Nurse Midwives
AWW: Anganwadi workers
ASHA: Accredited Social Health Activists
KIIs: Key informant interviews
FGDs: Focus group discussions
